# Autonomous Driver Based on an Intelligent System of Decision-Making

**DOI:** 10.1007/s12559-015-9320-5

**Published:** 2015-02-13

**Authors:** Michał Czubenko, Zdzisław Kowalczuk, Andrew Ordys

**Affiliations:** 1 Faculty of Electronics, Telecommunications and Informatics, Gdańsk University of Technology, 11/12 Narutowicza Street, 80-233 Gdańsk, Poland; 2 Department of Systems Engineering, Military Technological College, P.O. Box 262, PC 111 Muscat, Sultanate of Oman

**Keywords:** Artificial intelligence, Autonomous cognitive systems, Decision-making systems

## Abstract

The paper presents and discusses a system (*xDriver*) which uses an Intelligent System of Decision-making (ISD) for the task of car driving. The principal subject is the implementation, simulation and testing of the ISD system described earlier in our publications (Kowalczuk and Czubenko in artificial intelligence and soft computing lecture notes in computer science, lecture notes in artificial intelligence, Springer, Berlin, 2010, 2010, In Int J Appl Math Comput Sci 21(4):621–635, 2011, In Pomiary Autom Robot 2(17):60–5, 2013) for the task of autonomous driving. The design of the whole ISD system is a result of a thorough modelling of human psychology based on an extensive literature study. Concepts somehow similar to the ISD system can be found in the literature (Muhlestein in Cognit Comput 5(1):99–105, 2012; Wiggins in Cognit Comput 4(3):306–319, 2012), but there are no reports of a system which would model the human psychology for the purpose of autonomously driving a car. The paper describes assumptions for simulation, the set of needs and reactions (characterizing the ISD system), the road model and the vehicle model, as well as presents some results of simulation. It proves that the *xDriver* system may behave on the road as a very inexperienced driver.

## Introduction

For a long time, we can observe a great progress in the development of control theory and application of its results. More and more devices have built-in computers, which make some decisions. Robots entered schools, where children learn languages, solve tasks, etc., on the basis of some kind of cooperation with robots. For instance, specially designed robots replace humans in serious operations performed by firefighters or soldiers [6]. Robots supply also a vast help in medicine: ultra-precise surgical robots, cutting with greater precision than humans, assistants in rehabilitation, companions of human that deal with the elderly, disabled, children, etc. [5, 7, 42, 44]. The progress is a result of increasing autonomy of robots [27, 39]. There are also projects related to autonomous vehicles, aircrafts or vessels, which are able to decide on their moves [9]. Imitating this idea, there are systems that are able to decide when, what and how they learn about the objects or the behaviour of the environment [40].

As a general note on the development of mobile robotics, and based on the drive type, we can distinguish the following types of robots [36]:

- wheeled robots—used mainly for simple purposes (like line followers)
- tracked/crawler robots—which can move in sophisticated, or natural areas (for reconnaissance purposes, for instance)
- walking robots—operating in a specific advanced environment, both industrial and natural (used for various purposes)
- hybrid robot solutions.

In recent years, the branch of walking robots is increasingly developing. The ability to walk implemented in robots allows them to take the stairs or go through the rubble, etc. Among them there are spider robots with 6–8 legs (and many joints), which are stable but difficult to control, as well as humanoid robots having 2 legs, which are very difficult to control and keep stable.

But, what is more important, humanoid robots are received as more friendly and acceptable in society [16]. This approach is also used in the case of autonomous vehicles, where the cars have human features such as name and gender [46]. The idea of granting the appearance of a human to autonomous machines is becoming increasingly popular.

One of the examples of such projects, which implements both human appearance and features (to some extent), is a FLASH project [10, 17]. FLASH works on the principle of the inverted pendulum and is able to autonomously move and catch some items. It can also express emotions using a specially designed head. With the expression of feelings, it can better communicate with children. A robot EMIEW is similar [13], as it also operates on the principle of the inverted pendulum. Both robots are well suited to assist humans in various needs of life. A humanoid (certainly, to a certain extent only) robot called NAO, equipped with a medium-power on-board computer, is able to recognize various types of objects. Both NAO and its *grandfather*-ASIMO [41] can walk on two legs, in a very smooth way [24]. Such basic skills make the robots similar to human.

A good example, showing how much different humanoid robots have evolved, is the well-known DARPA Robotic Challenge, which is a competition of robots assisting humans in case of industrial disasters.

### Unmanned Ground Vehicles

A similar competition, which is intended for developing robotics and associated technologies, is the DARPA Grand Challenge, and in particular, its Urban version. Due to this competition, many autonomous vehicles and technologies related to them have evolved. The idea of unmanned ground vehicles (UGV) has a long history. The very first project of this kind was described in [11], where automobiles powered by electrical circuits and steered by radio were being engineered. It was though only a vision. Currently, several states and countries are introducing law regulations and highway code on the use of autonomous vehicles. Among them, the Google Car caused a sensation among people [28]. The scientists are thinking also about designing and creating cars controlled by the mind such as AutoNOMOS [12]. There are also concepts of a driver assistance using ideas derived from cognitive systems [14, 47], or augmented reality [34], for example. It cannot thus be doubted that this vision is becoming a reality now.

There are a great number of projects that develop the concept of unmanned vehicles [8, 25, 38]. Nevertheless, there are no real systems that outperform a human driver (who also is an imperfect system, but still the best one that is known). Many design methods are based on artificial intelligence, such as fuzzy systems, neural networks, evolutionary algorithms or rule-based methods.

**Fig. 1:**
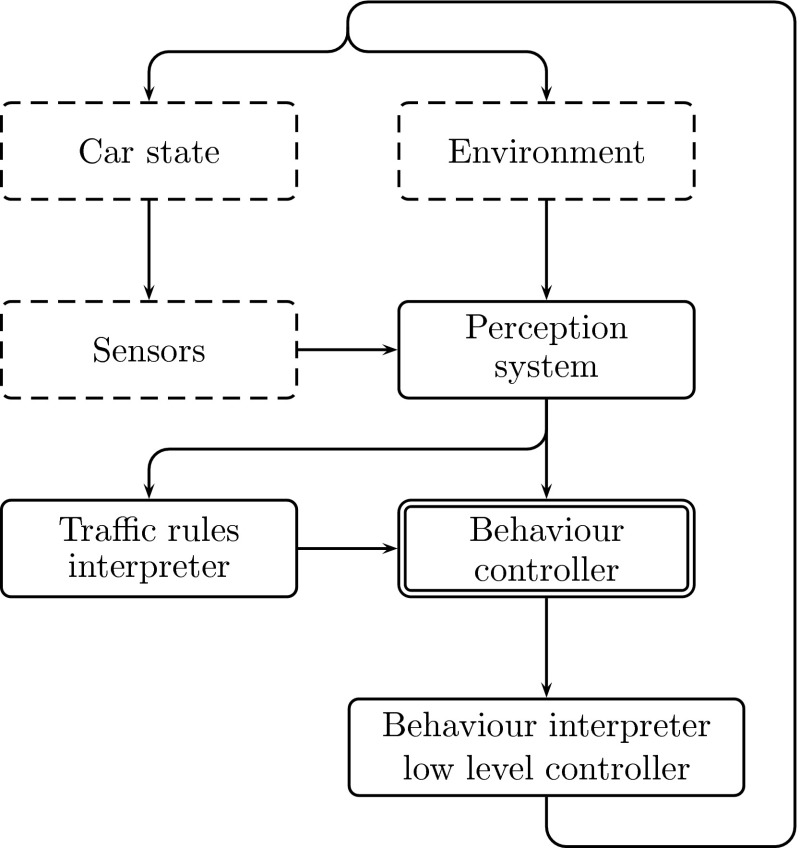
Abstract layer of an unmanned ground vehicle control system

The systems for autonomous driving are quite complex and can be divided into few subsystems (in abstract layers shown in Fig. 1):

- perception system
- traffic rules interpreter
- decision system (behaviour controller)
- low-level car controller.

The perception system corresponds to extraction of the abstract data (e.g. positions and types of surrounding objects) from the raw data. The data are perceived by sensors from the environment, both inner (car) and outer (road and its neighbourhood via camera, laser, etc.) sensors. The traffic rules interpreter checks the current rules (extracted from abstract data) and points at reactions that are compatible with them. The role of the decision system is to decide what reaction the system should perform (e.g. the ones compatible with rules or other reactions, like emergency braking). The chosen reaction is then translated to language expressions that steer actuators via a behaviour interpreter.

### Intelligent System of Decision-making

From the viewpoint of the evolutionary theory, mammals represent the highest form of life that has amazing auto-adaptation features (e.g. squirrels can adapt to changeable labyrinth tasks in order to get food). The highest species of mammals is *homo sapiens*. Therefore, the human decision-making system can be considered the most efficient of all known such systems and can serve as a standard for auto-adaptation. Taking into account cybernetics theories [30], which advise following nature in modelling objectives, a control system based on human psychology models is expected to be able to adequately perform the task of steering a car.

An Intelligent System of Decision-making (ISD), as presented in [19–23], can be such a system. It implements models of cognitive and personality (motivation) psychology for a control system of an agent [22]. It shows how people make a decision, from the incentives, to a reaction. The design of the whole ISD system is a result of a thorough modelling of human psychology based on an extensive literature study. This approach can thus also be used for controlling unmanned ground vehicles, including cars. The studies in this paper concern an adaptation of the ISD system to the role of an intelligent driver (*xDriver*), in a virtual environment.

The main goal of the *xDriver* system is not to create another instance of an autonomously controlled car, but to prove that a computational management system founded on the developed ISD model of human psychology is able to operate in a satisfactory manner in concrete critical conditions with numerous restrictions imposed on it. On another hand, the goal is also to assess whether the cognition-based control can be correctly performed in a way comparable to traditional control systems.

One of the modern control ideas of adaptation to changing conditions is the reconfigurable control approach [4, 35, 45, 51] that is based on the methods and procedures of diagnosis. Factors and diagnostic indicators, in a way similar to the early adaptation mechanism known as the technique of scheduling variable, allow one to take the right strategy very quickly [18]. Similarly, they may be applied in database systems designed for error detection and diagnostics [51]. In the case of ISD, the role of such indicators is played by certain ideas taken from fuzzy logics and linguistics (as they are applied in expert systems).

Such a concept, which implements the reconfigurable control approach and uses a fuzzy logic switching technique for autonomous navigation of the car, is applied in [1, 49, 50], where the low-level (trajectory) controller is based on a rule-fuzzy expert system.

The idea of variable system configuration, due to external conditions, can also be deduced from the cybernetics paradigm. In living organisms, the possibility of the reconfigurable approach to control is provided by emotions [2, 32]. For instance, emotions make an immediate and concrete basis for producing an effective control for an agent in hazardous conditions [3, 15]. Emotions can eliminate reactions that are locally or temporarily inefficient, and unclog other movements (known or formerly learned) ’most’ suitable for the situation. Thus, emotions provide the system with a higher level of autonomy [27, 39], which allows the system (agent) to choose the correct behaviour, even in the most difficult circumstances. In practice, various computational models of emotions can be of use in autonomous agents [37].

Emotions in the ISD system perform their function at a higher level of control than the basic ISD control ruled by the need system. In our robotics (agent) practice, they allow us to narrow down the set of possible reactions to the movements that are most adequate (in the view of the designer) for the current time moment and state of the ISD system [23].

Note that from the viewpoint of psychology, in particular, according to the human motivation theory, emotions are one of the most important elements of human behaviour. Systems, based on the model of human psychology (both cognitive and motivative), without emotions would be lame and totally inadequate. Actually, the presented experiment does not take into account the idea of emotions. This concept is mentioned here only as an integral part of the ISD. The whole idea of the higher-level control system (including emotions) is coherent and ready for implementation in future work.

**Fig. 2:**
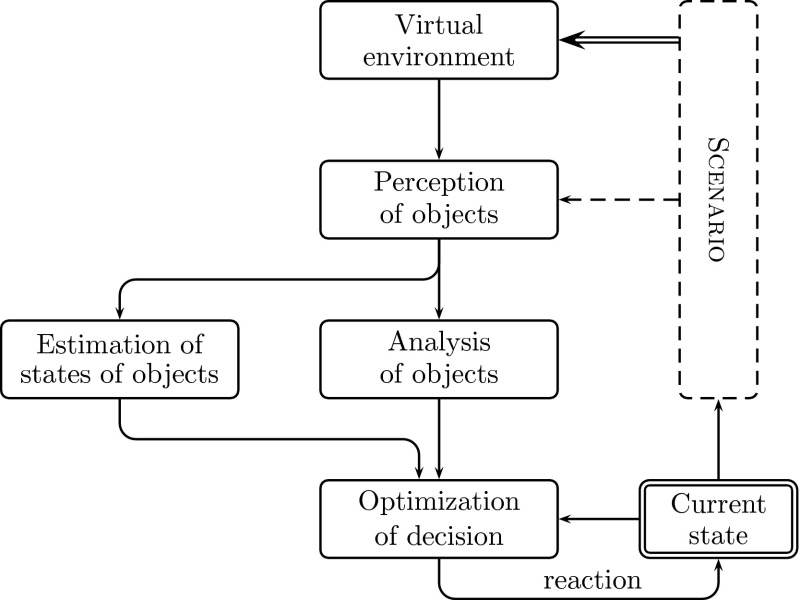
Block diagram of the ISD system after adaptation to the car driving task

## ISD System Adaptation

The driver model is thus strictly the ISD system with appropriate sets of reactions and needs, a working system of perception, connected to a road model. In its core mechanism (idea), the system has its own needs and tries to satisfy them, by reacting appropriately. Each reaction of the system influences the environment, which, in turn, provides feedback to the system. In order to use the ISD system as a driver, some minor changes are necessary to adapt it for such a task. The adaptation of the ISD system to the driver tasks is performed in three steps:

- integration of the perception systems with the simulated environment
- creation of an interpreter of traffic rules
- designing an adequate set of reactions and needs (*H*) according to emotional context (*ξ*).

The model of an adopted ISD is shown in Fig. 2. The environment is constructed on the basis of a certain scenario (which also takes into account the state of the car: position, velocity and acceleration). The *xDriver* can perceive objects in its view area (scene). The shape of the *xDriver* perception area strongly depends on the scenario, especially on bends and slopes of the road. The *xDriver* system computes the estimated position of objects, according to the state of the car and its current scene. Furthermore, the effects of the current traffic regulations and the objects in the view area can be assigned to the *xDriver* states (of all its needs *H* and emotion *ξ*). Thereby, the system can easily find the new reaction and put it into use. In return (as a feedback), the reaction affects accordingly the current state of the car.

Note that this model is a derivative of cognitive psychology, adapted for the purposes of the autonomous driver. It simply mimics the way in which the driver reacts to certain stimuli.

### Needs and Emotions

**Fig. 3:**
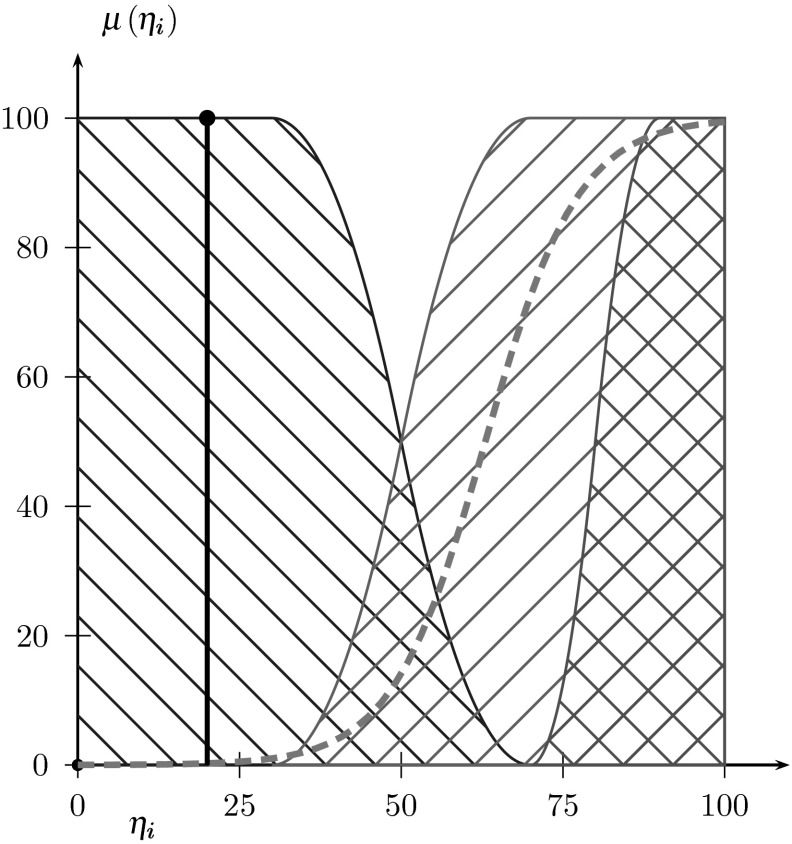
Exemplary fuzzy membership of an *i*-th need: the bold dashed line denotes the weighting function $(\omega (\eta _i))$, the (*blue*) sparsely hatched-backslashed area describes the satisfaction state, the (*red*) densely hatched-crossed area portrays the alarm state, and the (*green*) densely hatched-slashed area means the prealarm state; the thick vertical line marks an actual value of the unfulfilment degree *η*_i_ [20] (Color figure online)

Needs and emotions constitute a crucial part of the ISD model, since they describe the human motivational system. In the same way, they allow us to ’control’ the *xDriver*’s desire to act. The symbol *η* represents the degree of unfulfilment of a certain need and hereinafter *η* will be called a need (Fig. 3). It is an abstract fuzzy value, which takes one or more (two) of three states: satisfaction (lowest), prealarm and alarm (highest) [22]. It can, for instance, be partially satisfied and partially prealarmed (according to its actual crisp value). A need is completely satisfied whenever its crisp value is equal to zero (the applied negative logics is more suitable for several implementational reasons). All needs (*H*) are grouped according to their importance in a Maslow pyramid (5 levels) [29]. Moreover, each need of the same pyramid level has its individual importance, which changes according to its current state of satisfaction. This importance is described by a weighting function $(\omega (\eta _i))$ [22], which takes the form of a sigmoid curve. The inflection point of the function starts at the foot (beginning) of the membership function of the alarm. The weighting emphasizes the importance of alarmed needs. On this basis, it is easier for *xDriver* to choose those needs that require immediate reaction and fulfilment.

The number of the needs in such an abstract control system depends on the system designer (creator) and may be different than in human standards (note that a child has about 26 basic needs). In the case of *xDriver*, the system of needs has been simplified to seven needs, as follows:

- physiological (principal) level: energy optimization
- physiological level: goal achievement
- safety level: security of car
- safety level: traffic regulations
- (self-)esteem level: speed
- (self-)esteem level: confidence
- self-actualization level: creativity.

The energy optimization need corresponds to minimization of the quantity of the car manoeuvres. Each change of the speed of the vehicle (when braking, for instance) has an impact on this need: if the speed is changing, its respective quantity *η* is increasing (a corresponding positive/fulfilment level for this need would decrease) proportionally to the speed change. The need of goal achievement is connected to the travelled (virtual) section of the road. Its level of fulfilment wanes when *xDriver* recedes from the goal, and increases when it is getting closer to the goal. These two needs are at the bottom of the pyramid, as they are the most essential.

The purpose of safety (second) level of the pyramid is safe maintenance of the *xDriver* system. The security of the car is associated with accidents on the road (every little scratch reduces the fulfilment level of this need (increases its *η*), a major crash causes that this need goes to the highest state of alert). Furthermore, all traffic rules (including the speed restrictions) affect on the traffic regulations need. Any departure is sanctioned by waning its fulfilment level (in proportion to the quantity/importance of the departure).

The needs of the (self-)esteem (third) level are to counteract the safety needs and thus to promote counteraction over action; especially, the need of speed motivates *xDriver* to move faster. If the car is moving at a low speed (in terms of a fuzzy classifier), this need is growing (its satisfaction level is decreasing). The confidence need is satisfied in cases of correct driving (no accidents or crashes). Clearly, it is the part of the self-esteem of the driver that represents the experience of the driver and contributes to the safety of the whole system.

The speed need is highly arguable, but from the psychological point of view, driving at full speed gives an esteem for an agent. This need is obviously opposite to the need of security. Thus, the driver must search for a (Pareto) optimum, when it could drive fast and responsibly. It seems very human. Moreover, in terms of Maslow, the higher the position in the Maslow pyramid the lower the importance, and vice versa, the lower the level of the pyramid, the grater the significance of the need group. According to the applied design formula, the need for the speed will only be considered when all other needs are already fulfilled. Due to the fact that the reactions which can satisfy the need of speed can also decrease the satisfaction with respect to the security situation/need, they can thus be easily excluded from the use in inappropriate conditions. This is illustrated by the behaviour of the xDriver, presented further in the article.

The social level of the Maslows pyramid is omitted here. Self-actualization needs are on the top of the pyramid, one can think of them only when the lower level needs are suitably satisfied. The need for creativity (its degree of unfulfilment) is growing in the case of frequent (and tedious) sequences of actions, and it decreases when a new action is implemented.

An important element of the ISD system is an emotion engine. The emotion *ξ* of the *xDriver* is associated with several factors: the level of satisfaction of all needs, the emotional context of perceived objects and the earlier state of emotion. There are nine states (colours) of emotion: neutral, anger, anticipation, joy, trust, fear, surprise, sadness and disgust, each in three different amplitudes [33]. When emotion is not in a neutral state, it unlocks reactions that have emotional context (e.g. in case of surprise, the reaction of braking down is unlocked). The higher the magnitude of the emotion, the more desirable is a reaction (within its emotional context).

It is thus transparent that the system of emotions used in the xDriver allows for faster decision-making in emotionally detectable conditions (especially, hazardous or dangerous circumstances), for which there are feasible emotionally designable strategies. In addition, the use of a changeable database of possible reactions allows us to eliminate the possibility of taking a relatively dangerous reaction under safe conditions and vice versa. Simple examples which illustrate the effects of such unsuitable or excessive reactions are the manoeuvres of heavy braking or too-delicate braking. Under normal conditions, for example, performing a quick manoeuvre may threaten the safety of other road users. However, when the situation begins to endanger life, or in the face of other dangers, such as the intersection of the trajectories of two vehicles, a rapid manoeuvre may become necessary. Thus, after detection of a collision situation, the xDriver changes its emotional state and next unlocks the reaction of rapid braking and blocks other, not suitable reactions (like slow acceleration). Note that the emotional part is only described here, as a basis for future works.

### Reactions

The reaction is a sequence of simple and natural (for humans) movements (e.g. movement of the hand, or one step). However, you need more complex reactions when driving a car. The applied set of reactions of the *xDriver* include:

- increasing speed (by opening the throttle) to a set value
- decreasing speed (by closing the throttle) to a set value
- decreasing speed (by braking) to a given value
- changing lane to a selected direction
- switching the lights on/off.

Reactions such as emergency braking, avoiding obstacles, speeding up and others have an emotional context (can be used only under certain circumstances). The system is also capable of creating new reaction, as a sequence of basics reactions (e.g. an overtaking reaction should consist of reduction of gear, turning light signal on, changing lane, speeding up and changing lane one more time), in an evolutionary way, according to a learning scheme, or even at random. Other reactions, such as changing the gear, are planned to be added in future studies.

The implementation of a reaction is a single thread. The system waits for the effects of a current reaction, unless the perceived objects do interrupt. For instance, the overtaking reaction is implemented while the other car is not overtaken. It may be interrupted by changes in the current environment, for instance, by a car which shows up in front of the *xDriver*—in this case, the system can take one of the reactions of the current emotional context (pull back and return to its lane).

### Making the Decision

**Fig. 4:**
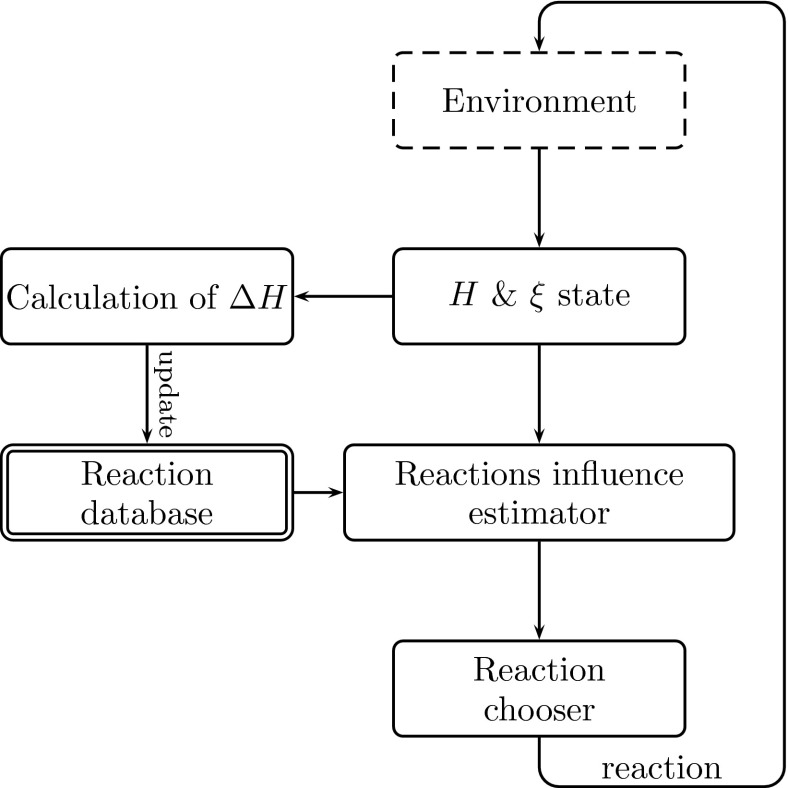
Algorithm for choosing the next reaction of the ISD

The block diagram of the algorithm that chooses reactions is shown in Fig. 4. **The environment** block is responsible for all objects near the road (the outer environment) and for the interpretation of the traffic rules corresponding to the perceived signs (the inner environment). Changes in the current environment of the *xDriver* influence **the states of** *H* **and** *ξ*. Namely, the observed objects have their concrete emotional and need context and a specific representation written in the *xDriver*’s memory, and these data have a definite impact on the system state. Some objects have strictly crisp values of need change (e.g. a pedestrian perceived on a zebra crossing adds 20 to the need for car security—as it endangers the car security and thus raises the degree of unfulfilment). Other objects (such as cars and signs) require the calculation of this impact in relation to the current parameters of the *xDriver*. For example, if the current speed of *xDriver* is about 100 km/h, and it perceives the sign of the recommended speed of 50 km/h, the impact of the traffic regulations need is calculated as $(100 - 50 )/\sigma$, where $\sigma \,=\,10$, thus the implemented need change equals five. A detailed description of this mechanism is presented in [19, 22, 23].

**Fig. 5:**
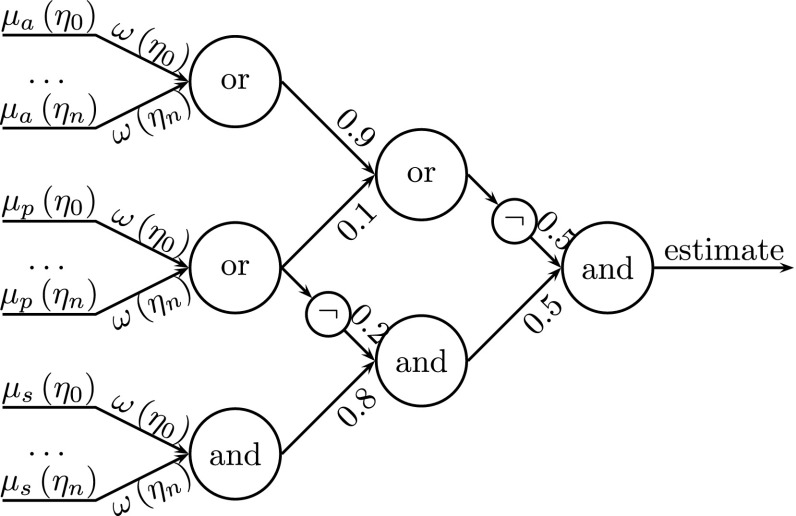
Fuzzy neural network estimating the suitability of reactions based on the actual state of needs and the simulated effects of reactions [19]: the inputs of neurons are (from the bottom) the values of the membership functions of satisfaction, prealarm and alarm for all the considered needs; the importance of the needs is underscored by using their individual weights/weighting functions *η*_i_; the output of the network is an estimate $\hat{if}$ of the impact (factor) of a given reaction on the state of the needs *η*_0_ through *η*_n_

After updating the state of the ISD system (Fig. 4), the *xDriver* calculates the **estimates** $(\hat{if})$ **of the impact factor** of prospective reactions. Selection of the reaction (the third-level filter in psychological terms) is performed in the actual emotional and perceptional context, as it is based on two discriminants: feelings (emotion) and objects assigned to each reaction. Moreover, each reaction has its own assigned effect on the system (*H, ξ*) described in terms of state increments (i.e. the difference between the state after the reaction implementation and the initial states, before applying this reaction). The reaction impact is estimated based on the history/effectiveness of the application of the reaction. The impact estimator $(\hat{if})$ of a reaction is computed by using the following fuzzy formula:

1 $$\begin{aligned} \hat{if} = (( \forall \eta \, are\, S ) {\texttt{AND}} \;   {\texttt{NOT}} \ (\exists \eta \, is\, P ))\ {\texttt{AND}} \\ {\texttt{NOT}} ((\exists \eta \, is\, P )\ {\texttt{OR}} \; (\exists \eta \, is\, A)) \end{aligned} $$

where *η* represents a need and {*S P A*} are the states of a need {satisfaction, prealarm and alarm}. It means that $\hat{if} = 1$ when all needs are satisfied and none of them is in the prealarm or alarm state after the considered reaction. Formula (1) is illustrated in Fig. 5. The first (lower) neuron reflects fuzzy operations between the function of the membership of the needs and the fuzzy satisfaction set $(\mu _s(\eta _i))$ using the respective weights $(\omega (\eta _i))$ of these needs. The second neuron considers the fuzzy prealarm set $(\mu _p(\eta _i)$ and the third neuron reflects the fuzzy alarm set $(\mu _a(\eta _i))$. Both use the fuzzy membership and the weightings. The operations AND and OR are fuzzy neurons using the Einstein norms, and NOT means negation in the Yager sense [26].

The reaction is chosen on the basis of Eq. (1), which is equivalent to the network shown in Fig. 5. However, the inputs to the network/equation are not the values of the reactions or the needs. In fact, each input is the probable effect of a considered/potential reaction on the indicated need *η*_i_ in terms of the particular *s*-, *p*-, *a*-membership function $\mu _x(\eta _i), x=s,p,a)$, computed from the current state of the need $\eta _i$ and a learned correction $\delta \eta _i$ in the need taken from the system database. According to this, the equation in fact predicts a potential future averaged fulfilment (impact factor $\hat{if})$ of all the needs (an aggregate state of the needs) after the hypothetical execution of the reaction. In other words, it describes how much the need system would be satisfied after the execution of the reaction.

The input weights are basically weights of certain needs. They are calculated upon the current need value, its parameters (describing the membership functions) and the Maslow pyramid level [20, 21]. The hidden layers weights were selected experimentally [22].

After computing all impact factors (for a current situation), the ISD simply **chooses the reaction** which has the highest value of $\hat{if}$. In the next cycle, the reaction parameters, namely the impacts of the reaction on the states *H* and *ξ*, are accordingly updated. For the purpose of updating the **reaction database**, **calculation** of the difference between the current and previous states of needs (when the reaction was initiated) is carried out. Certainly, the calculation takes into account the environmental influence (like the current evaluation of traffic condition). Mainly, the ISD system translates the current road conditions (detailed road signs and traffic conditions) to its own specific ’well-being’—to be more specific, to the system of its needs (one of the inner system states of the ISD, as described above). In such a way, the *xDriver* can consider future effects of its reactions.

## Road Model

The road model consists of abstract objects, which are represented as discoveries in the ISD system [21]. Each object has its own features (impressions), emotional context (may be neutral or null) and need context (also may be null). A set of such objects can easily describe a simple straight road. For complex models, a tree structure is necessary, where road intersections can be represented as nodes.

The whole road model is written in the XML language. For example, a single lane is described as follows:

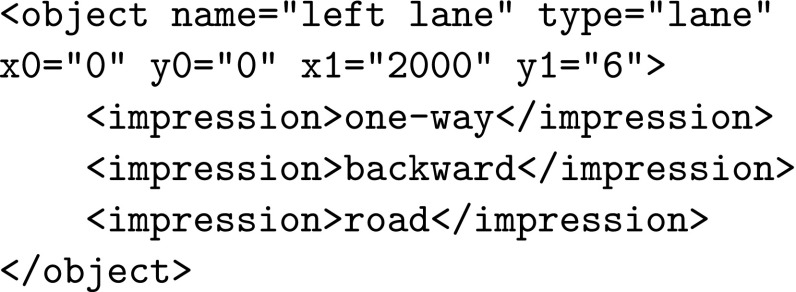



where the point (*x*_0_, *y*_0_) is a starting point, whereas the point (*x*_1_, *y*_1_) defines the end of the object. Features of the objects are treated as impressions. There are no strict rules of behaviour with respect to them. Thus, the car can drive the lane in opposite direction, even if the lane has the feature/impression ’one-way’.

In the case of the *xDriver* system, there are a few types of abstract objects:

- lane
- horizontal road sign
- vertical road sign
- virtual static objects (trees, houses, etc.)
- virtual dynamic objects (such as other cars, pedestrians, etc.).

Only the virtual objects listed above can influence the system (*H*, *ξ*). They can have a specific (not null) emotional or need context. Moreover, virtual objects have features, such as ‘hitable’ or ‘accidentable’, which point to certain possible consequences of interaction with these objects for the system.

## The Model of a Car

As an interface for the reactions of the *xDriver*, the car model should allow a wide range of inputs. And the car dynamics should be well defined. Such a model is described in [43]. Since the whole model is unnecessarily too complex for the purpose of testing the *xDriver* system, some modifications have to be applied to it.

First of all, it is assumed that the road is flat (no slopes). This simplifies the force of gravity to friction only:

2 $$\begin{aligned} F_{fric}=fmg \end{aligned}$$

where *f* = 0.012 is a friction coefficient, *m* = 2030 kg is the mass of the car, and *g* = 9.81 m/s^2^ is the gravity acceleration constant.

Another assumption concerning aerodynamic forces is that there is no external wind. Thus, the aerodynamic force can be presented as:

3 $$\begin{aligned} F_{air}=\frac{1}{2}\rho {}AC_{d}V^{2} \end{aligned}$$

where *ρ* = 1.226 kg/m^3^, *A* = 0.8, C_d_ = 0.32 is a coefficient dependent on the body of the car, and *V* is current velocity of the car. The engine force is simplified as follows:

4 $$\begin{aligned} \dot{V}=13.3*thr-0.3V\end{aligned}$$

5 $$\begin{aligned} F_{eng} = m \dot{V} \end{aligned}$$

where *thr* is a throttle position in cm, *V* represents velocity of the car, *and F*_eng_ is a drive force of the vehicle. Braking force is modelled by [43]:

6 $$\begin{aligned} \dot{F_{b}} = Ku - F_{b}/\tau \end{aligned}$$

where *τ* is a lag parameter, *Kc* is a pressure gain, and *u* is a pressure on a brake pedal. The transfer ratio between the steering wheel and the car wheels is defined as 1:16.

Such a model has few reaction inputs: throttle (in %), brake (in %) and steering wheel position. Thus, one can perform actions such as increasing or decreasing the velocity to a given value, braking to a given velocity value, changing position on the road and turning lights on/off. Moreover, the PI controller is used for steering the throttle position, for better performance of the system. The *xDriver* system is able to work also without the PI controller, but then the actions of increasing and decreasing speed would take too much time (the reason of such a behaviour is the inertia of the engine and the throttle pedal). The reactions can be interrupted by road signs (new in the view area), with velocity far away from the given value.

## Simulation Study

In order to prove that the *xDriver* system could take control over a virtual car in a virtual environment in terms of steering, accelerating and braking, a simulation study was preformed in the Java 7 programming environment with the use of external libraries (fuzzyj110a, jFreeChart, guava). The preliminary simulation used only one road, with ‘recommended speed’ signs, one zebra crossing and one lane narrowing. The *xDriver* had to drive to the goal, which was about 5,000 m ahead. The procedure of simulation is shown in Algorithm 1.

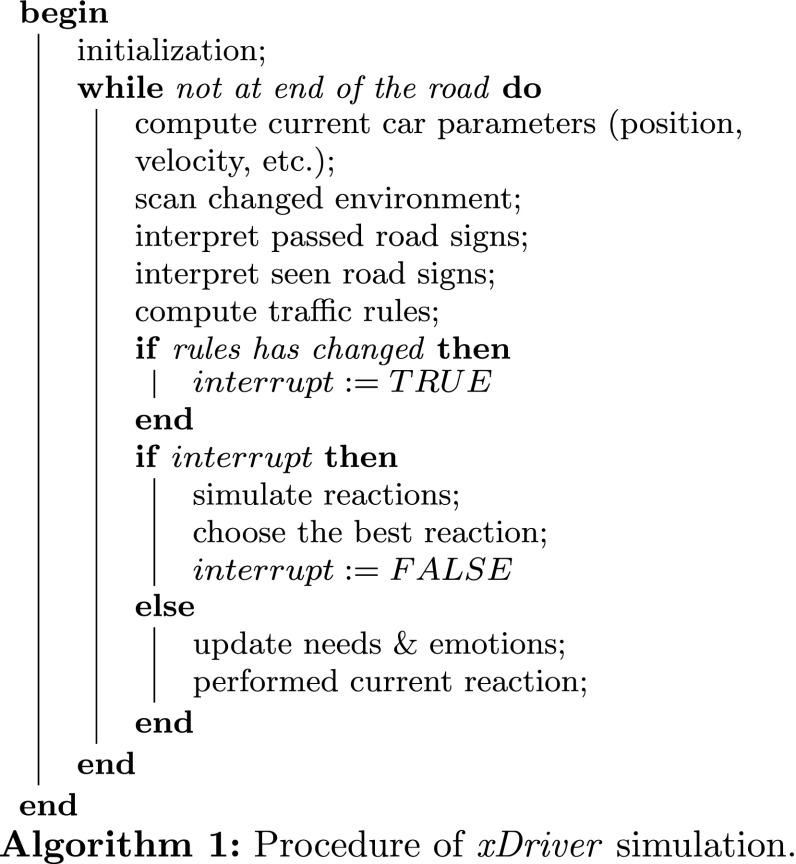



### The Scenario

The simulation scenario was relatively simple, and there were a few recommended speed signs and one command of lane change, without external objects. In the simulation, we relied only on the needs system (emotions were not involved). During the first part, the *xDriver* system acted like a cruise control system (CC), whereas the second part showed that the *xDriver* was something more than that. The road scenario is described in Table 1.

**Table 1:** Scenario of the simulation study, and the list of the *xDriver* decisions

Distance (m)	Scenario element
100	Recommended speed 90 km/h
1,000	Recommended speed 50 km/h
1,200	Zebra sign
1.400	Zebra
1.600	Cancel of 50 km/h
2,800	Recommended speed 30 km/h
3.200	Left lane order
3,400	Road narrows
3,800	End of road narrows
4,600	Right lane order
4,800	Cancel of 30 km/h

**Table 2:** List of the *xDriver* decisions

Distance (m)	Decision
0	Increment speed to 90.0
652.6	Decrement speed to 50.0
783.2	Keep current speed
850.1	Increment speed to 50.0
1,051.9	Keep current speed
1,163	Decrement speed to 50.0
1,173.3	Keep current speed
1,250.2	Increment speed to 90.0
2,054.7	Keep current speed
2,451.6	Decrement speed to 30.0
2,624	Keep current speed
2,651.9	Increment speed to 30.0
2,850.8	Change lane
3,214.6	Keep current speed
4,451.3	Change lane
4,535.4	Keep current speed
4,599.3	Increment speed to 90.0

In this particular scenario case, there was no need to use all of the needs, especially the *creativity* and *confidence* needs (Table 2) . The visibility range was set at the level of 350 m. Thus, decisions that were performed by the system were dependent on all signs in that range. Furthermore, the environmental influence on the needs was based on the difference between the allowed speed and the speed of *xDriver*, with an additional random value. There were also several rules that control the changes of the *xDriver*’s need as shown in Table 3. Even though the possible effects of reaction were estimated only with a certain probability, the system drove quite well. The resulting sequence of its decisions is given in Table 2.

**Table 3:** List of the rules of the environmental influences on the *xDriver*’s needs (the scale function calibrated values to the desired range (0–20) or (0–40), and all influences were contaminated by noise)

Rule condition	Need type	Influence
Keeping actual speed	Energy optimization	−2
Braking	Energy optimization	0.14
Approaching to the goal	Goal achievement	−((Distance to Goal)/5 − (100- goal achievement need))
Speed below the minimal	Traffic regulations	Scale(20, min − speed)/10
Speed below the minimal	Speed	−Scale(20, min − speed)/10
Speed is in traffic rules	Traffic regulations	−Random(0,10)
Speed is in traffic rules	Speed	Scale(20, max − speed)/10
Speed <70 km/h	Speed	Scale(40, max − speed)/10
Speed <70 km/h	Traffic regulations	−Scale(20, max − speed)/10
Correct lane	Security of car	−0.5
Incorrect lane	Security of car	5

The decisions made by the *xDriver* system were not repetitious (fuzziness used in the system and quasi-random factors cause such behaviour). What is more important, the reactions of increasing/decreasing the speed were not precise (even with the use of the PI controller), and they ended after the velocity went through the first oscillation which caused the undershoot/overshoot. In such cases, the *xDriver* oscillated around a certain speed (for example, after slowing to 50 km/h, it tried to keep the speed, but the throttle position was too low, so its subsequent reaction was to increase the speed to 50 km/h). This phenomenon was a clear disadvantage of the *xDriver* system.

To correct this problem, we do not need reactions of changing the speed, but transitions to a given speed value. Thus, the reactions should include only braking, changing speed to a given value and steering the wheels and gears (if they are allowed by the model). Moreover, we need a reaction which manipulates the attention of the driver, especially the so-called cognitive beam (a phenomenon of drawing attention by the objects from the road). The reactions should be able to expand and narrow the cognitive beam in case of perceived objects (such as informative road signs, objects on the road and built-up area). This issue will be studied in future.

**Fig. 6:**
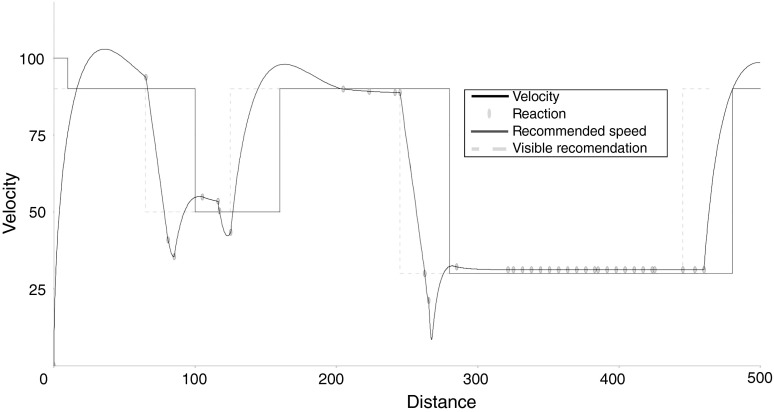
Velocity of the vehicle (*black continuous line*): the points of choosing a reaction (*yellow circles*), maximum recommended speed (*red continuous line*), recommended speed shifted due to the visibility range (*yellow dashed line*) all are functions of distance in decametres (Color figure online)

**Fig. 7:**
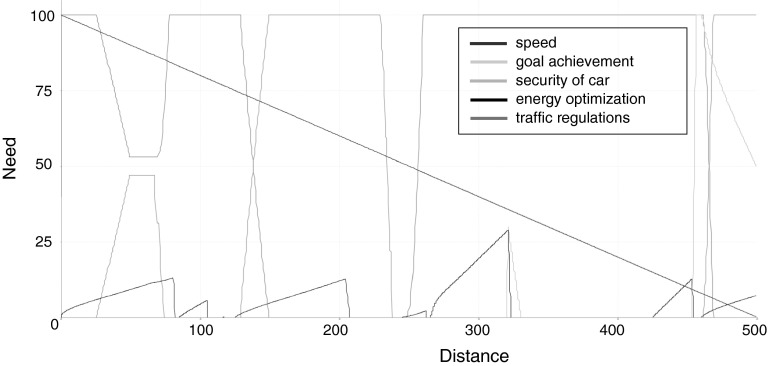
Crisp values of the *xDriver* needs, as function of distance (in decametres): the straight *blue line* represents the need for goal achievement, the blue trapezoid (line starting from the top) depicts the speed need, the energy optimization need is illustrated as a sawtooth *black line*, the red trapezoid line symbolizes the traffic regulation need, and the picks of the need for car security emerge around 3,200 and 4,500 m (Color figure online)

The results of simulation are shown in Figs. 6 and 7. The first reaction of the system was increasing the speed to 90 km/h. The reaction had been interrupted about 650 m, because the *xDriver* saw the speed limit sign. Thus, the next reaction was decreasing the speed to 50 km/h. In the next step, the *xDriver* tried to keep the speed at a certain level, but the inertia of the previous reaction was too large, so the speed was going down (the effect of an inexperienced driver). That caused the reaction of increasing the speed and oscillations around the speed of 50 km/h (due to consecutive repeated reactions).

When the *xDriver* saw the next road sign (the end of recommended speed), it increased the speed to 90 km/h and kept that speed during the next three reactions. Then, the oscillations showed up once again, due to the inertia of the previous reaction. The next reaction was changing the lane (at a given speed of −30 km/h) and series of reactions of keeping the speed followed. At the next point, after another lane change, the *xDriver* increased its speed to 90 km/h, according to the next sign (and with some delay).

Oscillations observed in the velocity curve (Fig. 6), resulting from the decisions of the xDriver, when driving on a straight road, are a typical behaviour of an inexperienced driver. In future studies, we would like to develop another variant of the xDriver, which would take into account the methods of machine learning, especially learning based on own mistakes (in this way, the oscillations should be eliminated). The presented simulation is a first attempt which proves that the system works correctly as an entity. We have not worked on the problem of obstacle avoidance, nor on the issue of optimization of the velocity curve. Also, driving on a winding and hilly terrain constitutes actually a very interesting challenge for the xDriver. Nevertheless, to implement this idea, the system would have to be substantially developed, taking into account a wide spectrum of possible road/driving scenarios. Note also that the emphasis of this restricted work is on modelling the driver and not on fully autonomous driving. Thus, the problem that concerns this case should be resolved in further research.

Interpretation of Fig. 7 is as follows. At a starting point, *xDriver* had two needs (of speed and goal achievement) in the alarm state, and one need (of energy optimization) rising from the satisfaction state. The degree of unfulfilment of the goal need decreased until the goal was reached. The energy optimization need went down when the *xDriver’s* speed did not change. When *xDriver* exceeded the recommended speed, its traffic regulation need (unfulfilment) started to increase and the value of the need of speed decreased. After it slowed down though, the behaviours of this two needs showed opposite trends. The recommended speed sign (noticed at 1300 m) and the reaction of *xDriver* changed the need for safety—traffic regulations (it started to rise to the top and stayed there until braking). Similarly, the next behaviour of *xDriver* was to change the lane—the speed need was completely unsatisfied, but the reaction of the system proves that the traffic regulation need was more important (*xDriver*’s speed was near to the recommended one). During the manoeuvre of lane changing, the need of car security started increasing. It happened due to the limited area of vision of *xDriver*, which resulted from travelling outside the lane.

Figure 6 shows that *xDriver* behaves like humans. It adheres to the speed regulations, though in a fuzzy way. To some extent, it is similar to cruise control (CC) systems. To adapt the *xDriver* system for the tasks of CC, we need to equip it with a different set of reactions and needs (allowing it to follow other cars, for instance).

**Fig. 8:**
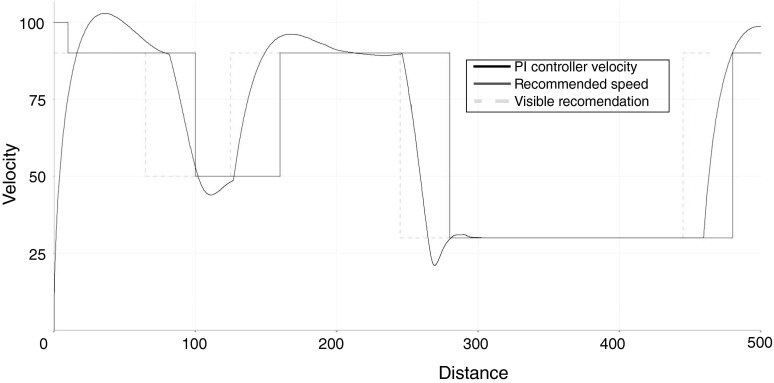
The velocity of the vehicle (*black continuous line*) steered by the PI controller, maximum recommended speed (*red wave line*) and recommended speed (*yellow dashed line*) shifted due to the visibility range, as functions of distance in decametres (Color figure online)

For comparison, another simulation was performed at the same scenario but with the use of a PI controller. It had the same configuration settings as the controller used in the *xDriver* in terms of the increasing/decreasing reactions (in response to perceived road signs). The PI controller parameters had been set manually ($k_p=0.4$, $K_i=0.4$, based on a MATLAB tuner). Probably, it would be possible to reduce the overshoot with a better PI tuning, but it was not the objective of this work. The results are shown in Fig. 8.

The curve of the speed for the PI-controlled system is more smooth—there are no drastic speed changes. On the other hand, *xDriver* reacts faster to the new road signs (on the basis of the velocity deduced by itself). In short, the *xDriver* behaved like an inexperienced driver. Supposedly, it can be trained like most people, to achieve better results.

## Summary

The presented paper proves that the Intelligent System of Decision-making (ISD) can perform the task of car driving. The model of cognitive psychology used in the ISD, especially the discovery concept, works out as a system that describes the road and its close environment. On the other hand, the motivation theory, especially the system of needs of *xDriver*, plays a crucial role in the task of creating autonomous ground vehicles founded on the ISD. The *xDriver* system is capable of driving correctly along an unknown road (having only the knowledge about the traffic rules). One disadvantage of the *xDriver* can be attributed to the implementation of reactions that are not most efficient in the sheer sense of the lower-level control system (where a common/PI controller could be better adjusted).

Moreover, the system does learn from the occurrences perceived—precisely, in the context of reactions and their impact on the system of needs and emotions (*H*, *ξ*). The context of environment is very important. The *xDriver* should learn also from the other users of the road and try to predict the traffic regulations (based on its earlier experiences). Nevertheless, the basic concepts have been proved, and the ISD system is able to manage the task of driving like a human.

In general, however, the *xDriver* project is still in its development stage. In fact, the present study considers only the basic behaviour and reactions of the *xDriver*. Hence, the lists of the needs and reactions presented here are relatively simplistic. Those lists can be further expanded as the system is developed. The research of more realistic situations and advanced systems will occur in the future (some works are already partly in progress). Such future work on the xDriver should apply, in particular, to more sophisticated road scenarios. It should consider, for instance, different road profiles, intersections and other road users.

Furthermore, the development of the idea of the *xDriver* system can also consider the use of:

- the multi-agents approach
- the cruise control system.

On the other hand, the mechanism of the ISD itself is also being continuously improved. Currently, the work on the memory model of ISD is in progress, where we consider a semantic network with the ability of forgetting, refreshing and restructuring the remembrances. The idea of a simple one-dimensional emotion is also under consideration.

From a practical viewpoint, it is worth mentioning that parallel to the development of the concept of ISD and the *xDriver* project, the ISD system is implemented on a NAO robot. Using the ISD, we intend to give this humanoid robot a sort of ’soul’. It is certain to have various needs, based on its own specific desire to act, emotions, memory (both semantic and episodic) and a composite set of reactions for the intended interaction with environment, including humans.
